# Image Texture Predicts Avian Density and Species Richness

**DOI:** 10.1371/journal.pone.0063211

**Published:** 2013-05-10

**Authors:** Eric M. Wood, Anna M. Pidgeon, Volker C. Radeloff, Nicholas S. Keuler

**Affiliations:** 1 Department of Forest and Wildlife Ecology, University of Wisconsin-Madison, Madison, Wisconsin, United States of America; 2 Department of Statistics, University of Wisconsin-Madison, Madison, Wisconsin, United States of America; University of Marburg, Germany

## Abstract

For decades, ecologists have measured habitat attributes in the field to understand and predict patterns of animal distribution and abundance. However, the scale of inference possible from field measured data is typically limited because large-scale data collection is rarely feasible. This is problematic given that conservation and management typical require data that are fine grained yet broad in extent. Recent advances in remote sensing methodology offer alternative tools for efficiently characterizing wildlife habitat across broad areas. We explored the use of remotely sensed image texture, which is a surrogate for vegetation structure, calculated from both an air photo and from a Landsat TM satellite image, compared with field-measured vegetation structure, characterized by foliage-height diversity and horizontal vegetation structure, to predict avian density and species richness within grassland, savanna, and woodland habitats at Fort McCoy Military Installation, Wisconsin, USA. Image texture calculated from the air photo best predicted density of a grassland associated species, grasshopper sparrow (*Ammodramus savannarum*), within grassland habitat (*R^2^* = 0.52, *p-value* <0.001), and avian species richness among habitats (*R^2^* = 0.54, *p-value* <0.001). Density of field sparrow (*Spizella pusilla*), a savanna associated species, was not particularly well captured by either field-measured or remotely sensed vegetation structure variables, but was best predicted by air photo image texture (*R^2^* = 0.13, *p-value* = 0.002). Density of ovenbird (*Seiurus aurocapillus*), a woodland associated species, was best predicted by pixel-level satellite data (mean NDVI, *R^2^* = 0.54, *p-value* <0.001). Surprisingly and interestingly, remotely sensed vegetation structure measures (i.e., image texture) were often better predictors of avian density and species richness than field-measured vegetation structure, and thus show promise as a valuable tool for mapping habitat quality and characterizing biodiversity across broad areas.

## Introduction

It is difficult to monitor and map patterns of wildlife diversity and abundance efficiently across broad areas. However, the need for doing so has never been more important given that approximately 12% of the world’s birds, 25% of its mammals, 40% of its amphibians, and 20% of its invertebrates are threatened by extinction [1], and trends in biodiversity loss are likely to continue [2]. In addition to directly monitoring biodiversity, ecologists routinely collect fine-grained information about habitat variables that may influence species diversity [3]–[5]. A common field-measured habitat metric used by ornithologists [6]–[9], and to a lesser extent, mammalogists [10] and entomologists [11], [12], is foliage-height diversity [3]. Foliage-height diversity is an index of vegetation structure that characterizes heterogeneity in vertical [3] and horizontal vegetation [7]. Variation in foliage-height diversity was originally used to predict avian diversity patterns and niche partitioning among species [3]. Since this seminal work by MacArthur and MacArthur, ecologists have linked foliage-height diversity to biodiversity in habitats around the world [7], [12]–[16]. However, although field-measured foliage-height diversity provides valuable fine grained information about habitat heterogeneity, it is logistically difficult to collect at large extents. This limits its use for management and conservation applications, which typically occur at broad-scales [17]–[19].

Remotely sensed data and especially land-cover classifications have been commonly used to monitor biodiversity across broad areas [20]–[23]. However, land-cover classes mask within-class variation in vegetation structure [24], [25]. This is problematic because variation in vegetation structure influences the distribution of biodiversity [3]. Remote sensing approaches that can characterize within-class vegetation structure include Light Detection and Ranging (LiDAR) [26], [27], Synthetic Aperture Radar (SAR) [28], and image texture [25]. Image texture, a measure of the spatial variation in image tone values, has been used to characterize vegetation patterns in heterogeneous habitats, including sparsely vegetated shrubland and desert [24], [29]–[31], grassland-savanna [25], and forest habitats [32], [33]. Image texture has also been used to predict species diversity (e.g., avian species richness) [24], [31], [34], habitat occupancy [32] habitat selection [35]–[37], and habitat suitability [38].

Image texture is a remotely sensed surrogate of vegetation structure and is valuable for ecological studies [25]. Yet, it is not clear how well image texture measures compare with indices of vegetation structure, derived from field-measured foliage-height diversity, in characterizing avian distribution patterns. Furthermore, the potential of image texture for predicting avian density (i.e., modeled abundance) is unexplored. This is important because density can offer insights into habitat quality [39] which is useful information for conservation applications. Additionally, the range of habitat types in which image texture effectively predicts avian species richness, a useful surrogate for biodiversity [40], is unclear.

Our goal was to explore the ability of image texture to predict and map high quality habitat and biodiversity among structurally disparate habitats. Our first objective was to assess the amount of variation in density of three avian species associated with a) field-measured foliage-height diversity and horizontal vegetation structure b) sample-point summaries of pixel values, and c) image texture measures from two remotely sensed data sources, a 1-m resolution infrared air photo and the Normalized Difference Vegetation Index (NDVI) derived from 30-m resolution Landsat TM imagery. Our second objective was to assess the amount of variation in avian species richness associated with the same three types of data. Our study was designed to examine the usefulness of image texture data for understanding and mapping fine scale patterns of avian density and species richness across broad spatial extents.

## Materials and Methods

### Study Area

**Figure 1:**
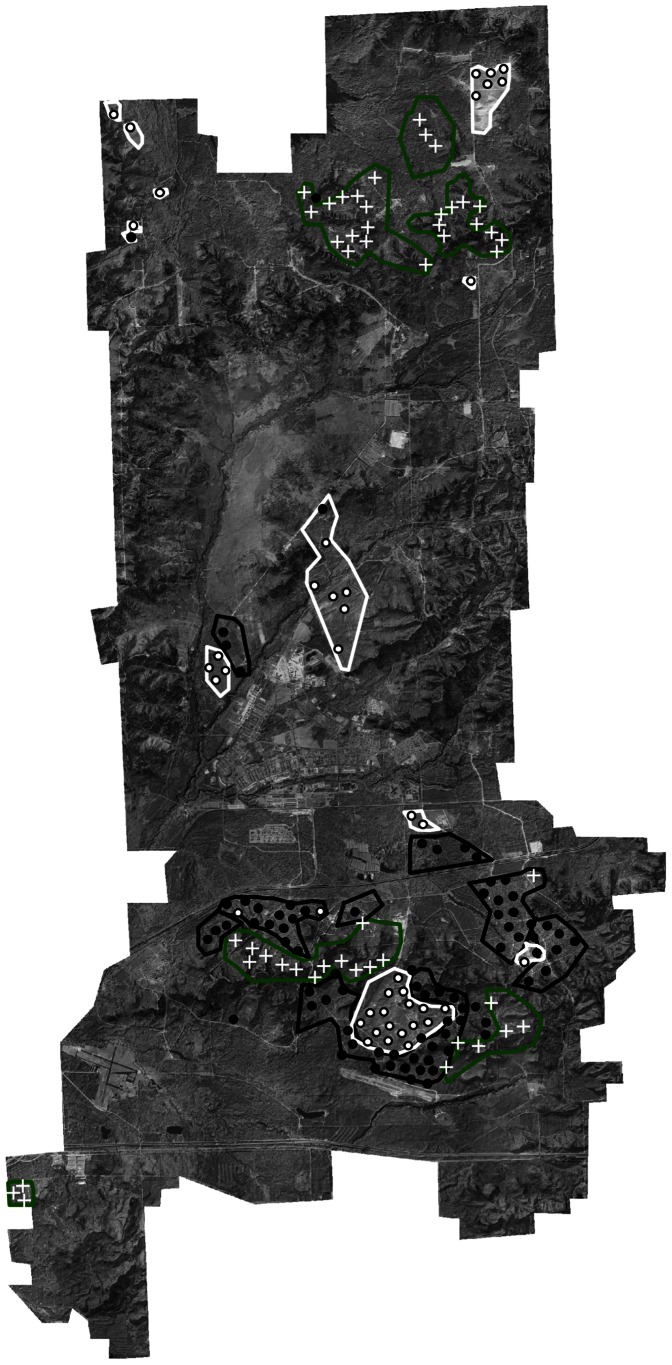
Distribution of 172 sample points at Fort McCoy Military Installation, Wisconsin, USA. White polygons indicate available grassland habitat with white circles denoting grassland sample points, black polygons indicate available oak savanna habitat with black circles denoting oak savanna sample points, and green polygons indicate available oak woodland habitat with white crosses denoting oak woodland sample points. A hillshade model calculated from a digital elevation model was set underneath a 40% transparent air photo to show topographical features of Fort McCoy.

We measured vegetation structure and avian abundance in the field across the 24,281 ha Fort McCoy Military Installation, in southwestern Wisconsin, USA (Fig. 1). Our supervisor while conducting field work was Timothy T. Wilder, who is the endangered species biologist for the Directorate of Public Works at Fort McCoy. Three habitat types occur within the boundaries of the available land for study. These include grasslands, which occur on 25% of the available land, and have less than 5% tree cover and low shrub cover; oak savanna (hereafter referred to as savanna) which occurs on 35% of the available land and is characterized by 5–50% tree canopy cover and variable shrub cover; and oak woodland (hereafter referred to as woodland), which occurs on 40% of the available land and is characterized by greater than 50% tree canopy cover and variable shrub cover (Fig. 1) [41]. Common tree species include, black oak (*Quercus velutina*), northern pin oak (*Quercus ellipsoidalis*), jack pine (*Pinus banksiana*), red oak (*Quercus rubra*), and white oak (*Quercus alba*). Shrubs include blueberry (*Vaccinium angustifolium*) and American hazelnut (*Corylus americana*), and the dominant grass is little bluestem (*Schizachyrium scoparium*).

To select field sampling points, we generated 400 random points, separated by at least 300 m, within grassland, savanna, and woodland habitat, using Hawth’s Tools [42] extension in ArcGIS 9.1 [43]. We identified the habitats from a leaf-on infrared air photo and a digital topographic raster map, created by Fort McCoy biologists. Texture calculations can be influenced by paved roads and other manmade structures. Therefore, we removed sample points within 150 m of such features from consideration. From this set, we retained sample points at least 100 m away from the edge of a focal habitat. Additionally, we only incorporated sample points if there was no significant disturbance (e.g., fire) between the dates when the remotely sensed data was acquired (see below) and when the field data was collected. This resulted in 172 sample points, 43 in grasslands, 78 in savannas, and 51 in woodlands (Fig. 1).

### Field-measured Vegetation Structure Measurements

**Figure 2:**
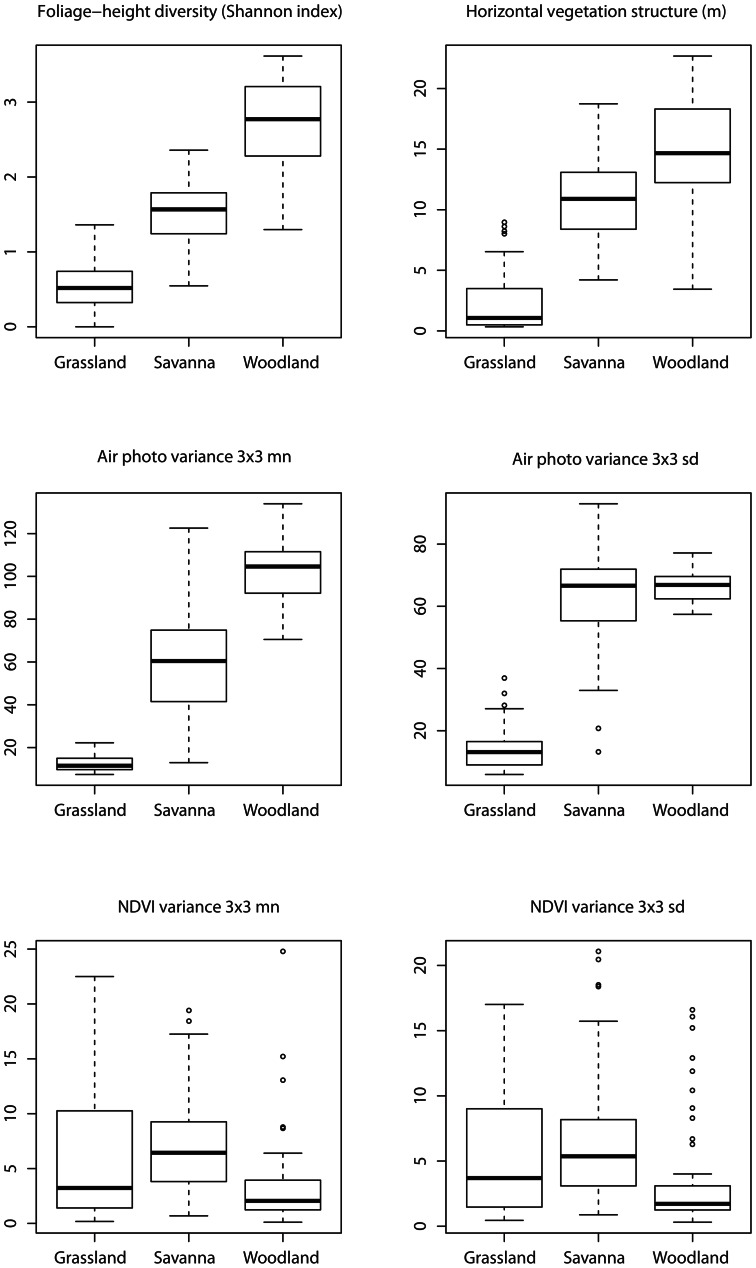
Box plot summaries of field-based vegetation structure, foliage-height diversity and horizontal vegetation structure, and four, remotely sensed, image texture measures of vegetation structure. First-order variance was calculated from a 1-m resolution infrared air photo and a 30-m resolution NDVI in a 3×3 window. Pixel values were summarized as the mean (mn) and standard deviation (sd) within a 100-m radius circle surrounding each of the 172 sample points. This figure was adapted and modified from [25].

At each sample point, we collected foliage-height profile measurements during the peak growing season from mid-June to late July in 2008 or 2009. Based on these measurements, we calculated foliage-height-diversity [3] and horizontal vegetation structure [7], using established methods [44]. We collected measurements at four 5-m radius sub-plots, located at the center of the sample point and with one each at azimuth angles of 0°, 120°, and 240°, at a random distance between 20 and 80 m so all foliage-height diversity measurements were entirely within the 100-m radius sample plot. We used the random distances to account for variation in vegetation structure heterogeneity among our focal habitats. At each sub-plot, beginning at the center-point, one observer walked 5 m in each of the four cardinal directions and placed a 12-m tall telescoping pole marked at 30-cm intervals vertically on the ground. A second observer recorded the number of instances where vegetation touched the pole (‘hits’) in each 30-cm section. If the canopy was taller than 12 m, then the second observer stood in an area where the view of the telescoping pole was not obscured by vegetation and used binoculars to estimate vegetation hits at the 30-cm intervals. This yielded 16 foliage-height profiles at each sample point (i.e., four measurements at each of the four sub-plots). From these 16 foliage-height tallies, we calculated two indices of vegetation structure. First, we computed foliage-height diversity using the Shannon diversity index [3], [25]. Second, we derived horizontal vegetation structure by taking the standard deviation of canopy heights at the 16 foliage-height diversity measurements per sample point [7]. Mean foliage-height diversity was 0.53±0.05, 1.51±0.05 and 2.70±0.09 in grassland, savanna and woodland, respectively (Fig. 2). Mean horizontal vegetation structure was 2.44±0.39, 10.89±0.36 and 14.65±0.67 in the three habitats (Fig. 2).

### Avian Point Counts and Indicator Species

At the 172 sample points, we conducted four 100-m variable radius, five-minute point counts from late May to early July in both 2007 and 2008 to characterize the avian community during the breeding season [45], [46]. In 2009 we visited sample points three times during the same time frame [47]. To distribute observer variability as equally as possible [46], four trained observers during 2007 and 2008 and three trained observers in 2009 performed one count at each sample point. Observers were extensively trained by the lead author on both bird identification and sampling protocol prior to field sampling, and the lead author was an observer each year.

**Figure 3:**
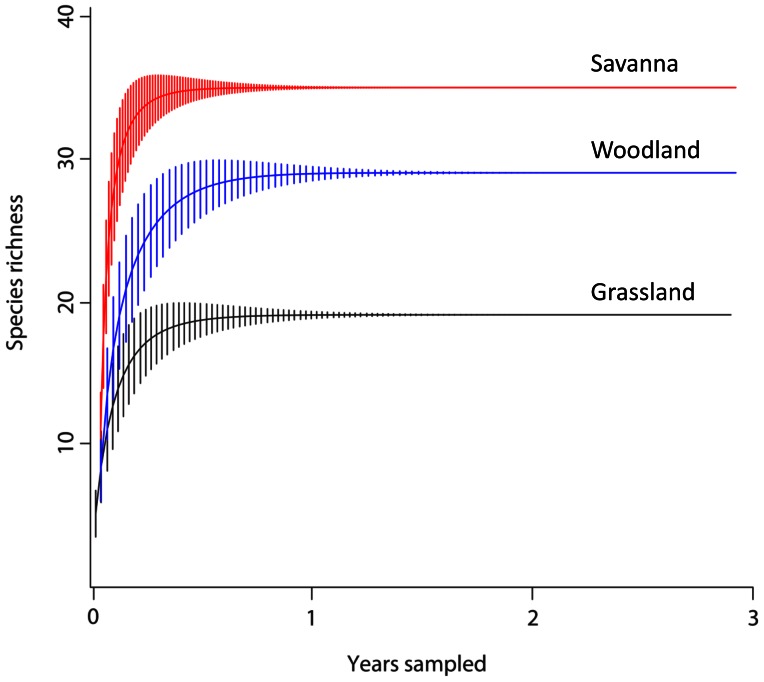
Species accumulation rarefaction curves, with standard deviation, for grassland, savanna, and woodland habitats across the three year, breeding season, sampling period.

We recorded abundance and distance estimates of three avian species, grasshopper sparrow (*Ammodramus savannarum*), field sparrow (*Spizella pusilla*), and ovenbird (*Seiurus aurocapillus*). We selected these birds as habitat indicator species based on their strong association with grasslands [48], savannas [49] and woodlands [50], respectively. Additionally, we calculated total species richness among all three field seasons for each sampling point. We used total species richness as our response variable because there was little species accumulation in each habitat throughout the three year sampling period (Fig. 3).

### Density Calculations

To reduce bias due to variations in species detectability, we estimated the density of the three avian species by adjusting abundance using Program Distance [51]. We fit six distance-adjusted models (half-normal cosine, half-normal hermite polynomial, uniform cosine, uniform simple-polynomial, hazard-rate cosine, and hazard-rate simple polynomial, [52]. We fit the six models for data from each species within their respective focal habitat for each year. We selected the global detection function from the top model using Akaike’s Information Criterion, which provided an estimate of sample-point specific species density for each year. We calculated the across-year average of the sample-point densities to obtain a robust measure of sample-point species density within each focal habitat. Vegetation structure, composition, and topography were similar within focal habitats. Because of this, we assumed that avian species detection probabilities were similar within habitats. Grasshopper sparrow detection probabilities in the three years were 0.51±0.06, 0.47±0.19, and 0.32±0.09 respectively, field sparrow were 0.39±0.07, 0.55±0.15, and 0.48±0.05, and ovenbird were 0.52±0.16, 0.55±0.20, and 0.51±0.13, and we calculated density based on these detection probabilities. We used the resulting sample point density estimates as dependent variables in statistical analyses.

### Remote Sensing and Image Texture Processing

Avian biodiversity has strong associations with vegetation productivity and greenness [53]. Green vegetation absorbs most of the solar radiation in the red wavelength, while reflecting about half of the near-infrared light [54], and variations in the ratio of infrared to near-infrared reflectance are associated with variation in vegetation productivity. Thus, we calculated sample-point pixel value (i.e., raw digital numbers) summaries from two sources of remotely sensed data that indicate productivity and greenness. The first was a 1-m resolution infrared air photo (hereafter air photo) taken on 25 August, 2006. Second, we used a 30-m resolution Landsat TM image acquired on 13 July, 2009 (path 25, row 29) from which we calculated the NDVI, which is a measure of photosynthetic capacity (greenness) [55]. Both images were captured during (Landsat TM) or just after (air photo) the peak of the growing season and thus corresponded with avian breeding conditions in our study area. Temperature and precipitation were similar among years [25] and the dominant trees (e.g., black oak) and shrubs (e.g., American hazelnut) in the focal habitats are slow growing [41]. Thus, the dominant structural features and vegetation greenness of the habitats, which influence reflectance values of the imagery, likely varied little throughout the study period.

Image texture calculations generate many measures that are collinear (Fig. S1) [24], [25], [34]. Thus, we used Wood et al.’s recommendations [25] to inform our selection of independent variables for predicting avian density and species richness. We included two first-order occurrence measures, variance and entropy, and one second-order measure, contrast [56]:

First-order variance:
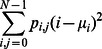



First-order entropy:
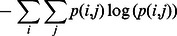



Second-order contrast:
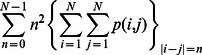



These three measures were previously shown to be strongly related with foliage-height diversity and thus we expected they would be useful for predicting bird density and species richness [25]. First-order texture measures do not consider the spatial arrangement of neighboring pixel values, while second-order measures do [56]–[58].

We computed the first-order measures, variance and entropy (i.e., Shannon diversity index) [56], with a moving window (e.g., a 3×3 window, see below), and the texture measure was assigned to the central cell of each window. To calculate second-order contrast, we summarized the pixel values within the moving window in a gray-level co-occurrence matrix (GLCM) and we calculated the texture statistic based on this matrix [56]. We calculated image texture using ENVI (Research Systems Inc., Boulder, CO). We used the tool ‘zonal statistics’ in ArcGIS 9.1 to summarize mean and standard deviation of sample-point pixel values and texture measure within 100 m of each sample point. Observed variation in image texture measures was not equal among habitats. For example, first-order variance, calculated in a 3×3 window from the air photo was highest in savanna and lowest in grassland and woodland (Fig. 2). On the other hand, first-order variance calculated in a 3×3 window from the NDVI was highest in grassland and savanna and lowest in woodland (Fig. 2).

Since the scale (as represented by a combination of the grain size of the image and the extent of the moving window) of an image texture measure may affect the strength of its relationship with avian density and species richness, we compared several window sizes for both image sources. We calculated image texture from the air photo in 3×3, 7×7, 15×15, 21×21, 31×31, and 51×51 1-m pixel moving windows (Table 1). We calculated image texture from the NDVI in 3×3, 5×5, 7×7, and 11×11 30-m pixel windows (Table 1). We chose these window sizes because they spanned the approximate territory sizes of the avian species, and captured information on the landscape surrounding each plot, which influence avian distribution patterns in grassland-savanna habitats [47].

**Table 1:** Description of imagery, including grain size, and the extent of the window size from which three image texture measures were calculated.

Imagery	Grain	Extent of window size
Infrared air photo	1 m	3×3, 7×7, 15×15, 21×21, 31×31, 51×51
Landsat NDVI	30 m	3×3, 5×5, 7×7, 11×11

The extent at which texture was calculated from the air photo ranged from 0.001 to 0.26 ha. The extent at which texture was calculated on the NDVI ranged from 0.81 to 10.89 ha. The extent of the 100-m radius circle where foliage-height profile measurements were collected, and sample-point pixel values and image texture were summarized (i.e., mean or standard deviation) was 3.14 ha. In Wisconsin grasslands, grasshopper sparrow territory sizes range from 0.32–1.34 ha [48]. In Illinois, field sparrow territories range from 0.31–1.62 ha [49]. Ovenbird territories range from 0.45–1.62 ha in Ontario, Canada [50]. Our window sizes and field-based data thus captured vegetation structure, sample-point pixel value summaries, and textural information across the reported range of territory sizes, in similar habitats, and geographically as close to our study area as possible, for the three avian species.

### Statistical Analysis

To investigate whether the amount of variation in avian density or species richness was best characterized by a) field-measured foliage-height diversity and horizontal vegetation structure b) sample-point summaries of pixel values, or c) image texture measures, we parameterized linear regression models with indicator species’ densities and avian species richness as dependent variables. For the density regressions, we only used data from within the indicator species’ habitat, while for models involving species richness we used data from all 172 sample points. We assessed the linear model assumptions of heteroscedasticity by fitting residual versus fitted values plots, and we visually examined if the spread of points were constant. We assessed normality by fitting *QQ-*norm plots and the linearity of residuals for each model by visually inspecting scatterplots. If necessary, we applied square-root transformations for the response variables, which were count derived density estimates, and we log transformed the independent variables. If model assumptions were met, but there was a lack of a linear relationship, we fit second-order polynomial regression models by adding a quadratic term.

To evaluate the predictive ability of the best fitting models (i.e., the models with the highest coefficient of determination), we used leave-one-out cross-validation calculated using the ‘boot’ package in program R [59]. We used the leave-one-out approach as opposed to k-fold cross-validation because it performs better when the number of observations is low and we had <80 sample points within each focal habitat. To check for spatial-autocorrelation among sampling points, we fit semivariograms of the residuals for the models for each indicator species’ adjusted density patterns and the models of overall avian species richness [60]. Semivariograms revealed no spatial autocorrelation affecting the models of either indicator species density or avian species richness. All statistical analysis was completed using the R software package [61].

## Results

### Predictions of Avian Species Density

**Figure 4:**
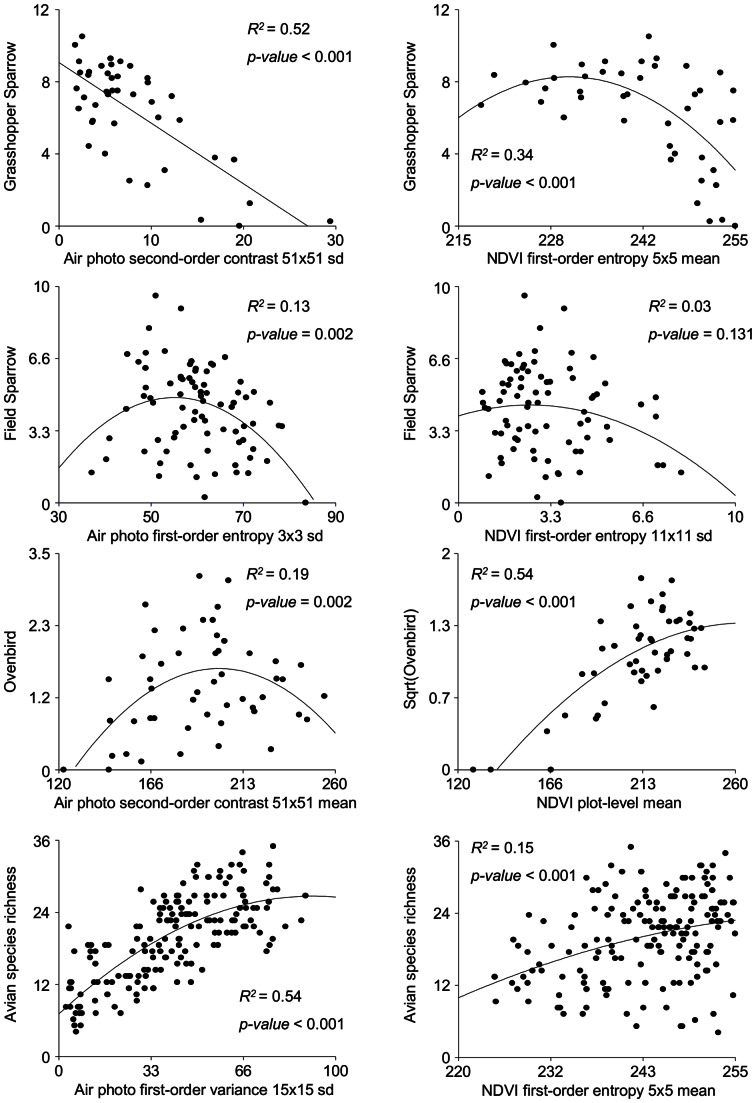
Scatter plots of the relationship between density of grasshopper sparrow at 43 grassland sample points, field sparrow at 78 savanna sample points, ovenbird at 51 woodland sample points, and avian species richness at all 172 sample points with texture measures derived from an infrared air-photo (left column), and NDVI (right column). All relationships significant at the *p* = 0.05 level except for field sparrow regressed against NDVI texture measures. The black lines represent results from linear regression with least-squares fitted and 2^nd^ order polynomial lines.

**Figure 5:**
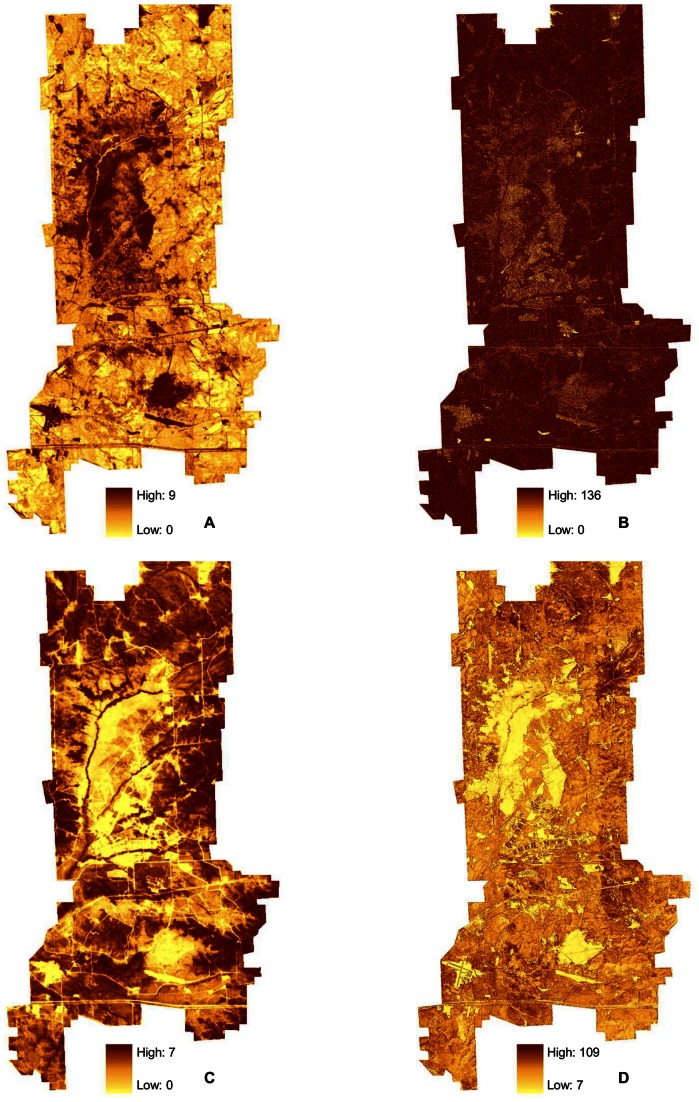
Predictive maps for density of three birds and avian species richness. Map A) represents grasshopper sparrow density, B) field sparrow density, C) ovenbird density, and D) avian species richness. Best model obtained from linear regression analysis relating density and avian species richness versus sample-point summaries and image texture measures calculated from a black-and-white infrared air photo and a NDVI. Equations used: grasshopper sparrow: y = 9.22+ second-order contrast 51×51 sd*−0.36; field sparrow: y = −10.85+ first-order entropy 3×3 sd*0.57+ first-order entropy 3×3 sd*−0.0052∧^2^; ovenbird: y = −4.7+ NDVI sample-point mean*0.05+ NDVI sample-point mean*−0.0008∧^2^; avian species richness: y = 7.08+ first-order variance 15×15 sd*0.40+ first-order variance 15×15 sd*−0.0021∧^2^.

Mean grasshopper sparrow density among the grassland sample points was 6.38±0.42. Grasshopper sparrow density was not significantly related to either foliage-height diversity or horizontal vegetation structure (Table 2). The sample-point pixel value mean summary of the air photo explained 26% of the variation in grasshopper sparrow density (Table 3). However, sample-point pixel value summaries of Landsat NDVI were not significantly related to grasshopper sparrow density (Table 3). Grasshopper sparrow density was most strongly related to the standard deviation of second-order contrast calculated from the air photo in a 51×51 moving window (*R^2^* = 0.52, *p-value* <0.001, Table 4, Fig. 4). The texture measure calculated from the NDVI that best predicted grasshopper sparrow density was the mean of first-order entropy calculated in a 5×5 moving window (*R^2^* = 0.34, *p-value* <0.001, Table 5, Fig. 4). The top model had a prediction error of 3.77 and the average prediction error for the air photo models was 5.32 (Table 4, Fig. 5).

**Table 2:** Results of linear regression analysis relating grasshopper sparrow, field sparrow, and ovenbird density and avian species richness to foliage-height diversity and horizontal vegetation structure.

Field-measured vegetation structure	*Adjusted R* ^2^	*p-value*	Prediction error
**Grasshopper sparrow**			
Foliage-height diversity	0.04	0.146	
Horizontal vegetation structure	0.06	0.060	
**Field sparrow**			
Foliage-height diversity	−0.01	0.774	
Horizontal vegetation structure	0.00	0.293	
**Ovenbird**			
Foliage-height diversity	0.10	0.009	0.74
Horizontal vegetation structure	−0.02	0.636	
**Avian species richness**			
Foliage-height diversity	0.32	<0.001	47.32
Horizontal vegetation structure	0.40	<0.001	39.62

The prediction error for significant models resulting from leave-one out cross validation is presented for significant models.

**Table 3:** Results of linear regression analysis relating grasshopper sparrow, field sparrow, and ovenbird density and avian species richness to 1-m air photo and 30-m NDVI sample point pixel value mean (MEAN) and standard deviation (SD) summaries.

Pixel value summary	*Adjusted R* ^2^	*p-value*	Prediction error
**Grasshopper sparrow**			
Air-photo MEAN	0.26	<0.001	6.13
Air-photo SD	0.01	0.280	
NDVI MEAN	0.13	0.051	
NDVI SD	−0.02	0.524	
**Field sparrow**			
Air-photo MEAN	−0.01	0.609	
Air-photo SD	0.04	0.072	
NDVI MEAN	0.02	0.195	
NDVI SD	−0.02	0.731	
**Ovenbird**			
Air-photo MEAN	0.00	0.339	
Air-photo SD	−0.02	0.580	
NDVI MEAN	0.54	<0.001	0.43
NDVI SD	0.16	0.006	0.53
**Avian species richness**			
Air-photo MEAN	0.04	0.008	46.27
Air-photo SD	0.35	<0.001	37.56
NDVI MEAN	0.13	<0.001	41.74
NDVI SD	0.00	0.598	

The prediction error for significant models resulting from leave-one out cross validation is presented for significant models.

**Table 4:** Results of linear regression air photo analysis relating grasshopper sparrow, field sparrow, and ovenbird density and avian species richness to image texture measures calculated within moving windows of six extents.

Texture measure	3×3	7×7	15×15	21×21	31×31	51×51	*p-val.*	Prediction error
**Grasshopper sparrow**								
Entropy MEAN								
Entropy SD						**0.17**	0.010	7.10
Variance MEAN	0.17	**0.23**					0.002	5.97
Variance SD	**0.46**	0.40	0.37	0.36	0.35		<0.001	4.44
Contrast MEAN								
Contrast SD	0.48	0.43	0.42	0.43	0.45	**0.52**	<0.001	3.77
**Field sparrow**								
Entropy MEAN	**0.10**	0.10	0.09	0.08			0.007	3.70
Entropy SD	**0.13**						0.002	3.82
Variance MEAN	**0.12**	0.09	0.08	0.08	0.08		0.003	3.85
Variance SD		**0.07**					0.021	3.66
Contrast MEAN	**0.09**	0.09	0.09		0.09	0.09	0.010	3.95
Contrast SD								
**Ovenbird**								
Entropy MEAN								
Entropy SD								
Variance MEAN	**0.17**	0.08					0.004	0.81
Variance SD								
Contrast MEAN	0.17	0.18	0.18	0.18	0.18	**0.19**	0.002	0.80
Contrast SD	**0.09**						0.028	0.76
**Avian species richness**								
Entropy MEAN	0.35	**0.42**	0.39	0.36	0.33	0.31	<0.001	44.69
Entropy SD	**0.41**	0.22	0.21	0.18	0.13		<0.001	47.73
Variance MEAN	**0.45**						<0.001	35.76
Variance SD	0.33	0.48	**0.54**	0.52	0.49	0.42	<0.001	23.20
Contrast MEAN								
Contrast SD								

Values within cells are adjusted *R* ^2^. Shown are the best model *p-value* (*p-val.*) and the prediction error for the best model (in bold), resulting from leave-one out cross validation. Cells not populated with metrics indicate non-significant models or assumptions of linear models (i.e., heteroscedasticity) could not be met.

**Table 5:** Results of linear regression NDVI analysis relating grasshopper sparrow and ovenbird density and avian species richness to image texture measures calculated within moving windows of four extents.

Texture measure	3×3	5×5	7×7	11×11	*p-value*	Prediction error
**Grasshopper sparrow**						
Entropy MEAN		**0.34**	0.29		<0.001	6.01
Entropy SD						
Variance MEAN						
Variance SD						
Contrast MEAN						
Contrast SD						
**Ovenbird**						
Entropy MEAN		0.13	0.13	**0.20**	0.002	0.73
Entropy SD						
Variance MEAN	0.09	**0.29**	0.26	0.27	<0.001	0.65
Variance SD		**0.19**	0.16		0.002	0.69
Contrast MEAN	0.12	0.17	0.16	**0.24**	<0.001	0.48
Contrast SD		0.09	**0.11**	0.09	0.026	0.60
**Avian species richness**						
Entropy MEAN		**0.15**	0.14		<0.001	40.56
Entropy SD		**0.09**	0.07		<0.001	43.24
Variance MEAN						
Variance SD				**0.09**	<0.001	48.36
Contrast MEAN						
Contrast SD						

Field sparrow model metrics are not displayed because no significant relationships found. Values within cells are adjusted *R* ^2^. Shown are the best model *p-value* (*p-value*) and the prediction error for the best model (in bold, highest *R* ^2^), resulting from leave-one out cross validation. Cells not populated with metrics indicate non-significant models or assumptions of linear models (i.e., heteroscedasticity) could not be met.

Mean field sparrow density among the savanna sample points was 4.29±0.21. Field sparrow density was not significantly related to vegetation structure indices (Table 2), sample-point pixel value summaries from either the air photo or NDVI (Table 3), or texture measures calculated from NDVI (Table 5). Field sparrow density was most strongly associated with the standard deviation of first-order entropy calculated from the air photo in a 3×3 moving window (*R^2^* = 0.13, *p-value* = 0.002, Table 4, Fig. 4). The top model of field sparrow density had a prediction error of 3.82. This was slightly higher than the best prediction error of 3.66 for the model fit using the standard deviation summary of first-order variance in a 7×7 moving window. However, this model captured only 7% of the variance in field sparrow density. The average prediction error for the air photo models was 3.80 (Table 4, Fig. 5).

Mean ovenbird density among the woodland sample points was 1.38±0.13. Ovenbird density was weakly related to foliage-height diversity (*R^2^* = 0.10, *p-value* <0.009), and not to horizontal vegetation structure (Table 2). The top model explaining ovenbird density was the sample-point pixel value mean summary of NDVI (*R^2^* = 0.54, *p-value* <0.001, Table 3, Fig. 4). The mean summary of second-order contrast in a 51×51 moving window calculated from the air photo explained 19% of the variance (Table 4, Fig. 4). Whereas the mean summary of variance calculated from the NDVI in a 5×5 moving window explained 29% of the variance in ovenbird density (Table 5). However, the mean summary of contrast calculated in an 11×11 moving window from the NDVI had the best prediction error rate of 0.48 among the Landsat models (Table 5). The overall top model had a prediction error of 0.43 and the average between the mean and standard deviation pixel value summaries of NDVI was 0.48 (Table 3, Fig. 5).

### Predictions of Avian Species Richness

Foliage-height diversity was moderately associated with avian species richness (*R^2^* = 0.32, *p-value* <0.001, Table 2). Horizontal vegetation structure was the best field-measured vegetation structure index explaining avian species richness (*R^2^* = 0.40, *p-value* <0.001, Table 2). The sample-point pixel value standard deviation summary from the air photo explained 35% of the variation in avian species richness (Table 3). Whereas, the pixel value mean summary only explained 4% of the variation in avian species richness (Table 3). The mean sample-point summary from the NDVI explained 13% of the variation in avian species richness (Table 3). The NDVI-derived standard deviation sample-point summary was not related with avian species richness (Table 3). Avian species richness was best predicted by the standard deviation of first-order variance calculated from the air photo in a 15×15 moving window (*R^2^* = 0.54, *p-value* <0.001, Table 4, Fig. 4) and NDVI-derived texture measures were only weakly associated with avian species richness (Table 5). The top model based on the highest coefficient of determination had a prediction error of 23.20 and the average prediction error for the air photo models was 37.85 (Table 4, Fig. 5).

## Discussion

Surprisingly, we found that image texture measures, and to a lesser extent, sample-point summaries of pixel values, were more strongly related to variation in avian density and species richness than field-measured foliage-height diversity and horizontal vegetation structure. This is an exciting advance and a significant step forward in the ability to map avian density and species richness over broad spatial extents. Effective methods for monitoring and mapping species distributions require broad-scale data, and remotely sensed data can provide an overview of habitat across extensive areas. However, the challenge is how to accurately characterize fine-grained wildlife habitat from remotely sensed imagery. We found that image texture predicts density patterns of avian species associated with grassland and woodland habitats well. However, this relationship was far weaker within savanna habitat.

In all cases, models fit using image textures measures, and for the ovenbird, the sample-point pixel value mean summary of NDVI, performed better than field-measured foliage-height diversity and horizontal vegetation structure in predicting avian density. Grasshopper sparrow density was highest in areas where second-order contrast was low in air photo and NDVI data. These low values correspond to the central areas of two large grassland patches that are devoid of large shrubs or trees. Grasshopper sparrows use large, open grasslands with little woody cover [62]–[64]. The north-central grassland patch at Fort McCoy is located within a non-accessible area (i.e., used for military training). This area was predicted to support high grasshopper sparrow density highlighting a useful conservation application of image texture in delineating good quality habitat in hard to reach or remote locations. Furthermore, the strongest relationship with air photo-derived data occurred at the broadest scale, 51×51 1-m pixels, (0.26 ha.) and the strongest relationship with 30-m NDVI data occurred at the 5×5 window scale (2.25 ha). Thus, texture measures derived from data sources that differ markedly in resolution, were both strongly associated with variation in grasshopper sparrow density at scales that span the species’ breeding territory size [48].

We found both field-measured vegetation structure indices and remotely sensed image texture were poor predictors of field sparrow density patterns. Field sparrows use habitats with sparse canopies and moderate to high shrub cover [49]. We expected image texture would capture the variability of tree cover within savanna habitats, where field sparrow were found in high densities, because image texture has been successfully used to characterize avian diversity in the sparsely vegetated Chihuahuan desert [24], [31]. While both field-measured and remotely sensed measures of vegetation structure are different in savannas than grassland or woodlands [25], this component of habitat by itself was not strongly associated with field sparrow density. The observed variations in both field based and, especially, image texture calculated from the air photo and NDVI were largest in the savanna habitats (Fig. 2). Because of this, it is likely that features of the savanna habitat that the Field Sparrow was responding to, such as vegetation composition or fine- scale habitat elements (e.g., herbaceous composition or downed woody debris) [5], [65], [66], were not captured by either the field-measured vegetation indices or image texture measures. Thus, we urge caution in applying image texture to discriminate high quality habitat in sparsely vegetated areas since the variation in predictor variables can be high, resulting in poor fitting models.

We found the sample-point mean summary of NDVI values was the best predictor of variation in ovenbird density. In Michigan forests, NDVI was a good predictor of ovenbird occurrence [67]. As we expected, ovenbird density was highest in locations with high NDVI which corresponded to dense, interior woodland. Furthermore, image texture measures calculated from the air photo explained up to a third of the variation in ovenbird density. Thus, we suggest that image texture can map high quality woodland and forested habitat well, especially when combined with other remotely sensed data (e.g., NDVI).

An unexpected finding of our study was the importance of matching the grain size of an image with the resolution of habitat heterogeneity (i.e., vegetation structure) within a habitat patch. Two habitats at Fort McCoy, grassland and woodland, occur in large, contiguous patches. Therefore, information generated using the 30-m resolution NDVI was moderately successful in predicting grasshopper sparrow (although not as strong as image texture calculated from air photo) and ovenbird density. Savanna habitats at Fort McCoy typically occur in smaller patches at the edge of grasslands or woodlands, and we were not able to find significant relationships between image texture calculated from NDVI and field sparrow density. Therefore, we speculate that the within-habitat variability of savanna habitats at Fort McCoy was difficult to capture using image texture measures calculated from the coarser grained NDVI. Thus, it is likely that the influence of the surrounding grassland or woodland habitat captured by the scale of the NDVI image texture measures (i.e., grain size and window extent) may have impacted the ability to predict field sparrow density. This is an important finding for conservation applications suggesting sample-point summaries and image texture from NDVI may be better at capturing variation in habitat that occurs in large continuous blocks, and not as well suited to assess habitat that occurs in relatively small patches.

The density of organisms can provide important information about habitat quality [39]. However, predictions of animal densities typically use field-measured data for unique habitat types [68], rather than remotely sensed data [69]. An advantage for conservation applications is that remotely sensed data can predict avian density patterns for large areas. However, previous maps of avian habitat were generated based on broad land-cover classes which omit important within-in habitat heterogeneity (e.g., vegetation structure). Based on our findings, and those of others [24], [31], [34] image texture data can provide a significant increase in the amount of information (i.e., broader coverage than field-measured variables) and spatial detail (i.e., heterogeneity of vegetation structure), which is necessary for broad-scale management and conservation planning.

This empirical finding is well supported by ecological theory, which postulated that higher vegetation structural diversity is associated with avian diversity [3]–[5]. We chose our study area because of its wide variation in vegetation structure diversity among habitats [25]. Foliage-height diversity and horizontal vegetation structure were indeed positively associated with avian species richness and accounted for 32% and 40% of the variance, respectively. Yet, the ground measured vegetation structure explained less variation in avian species richness than the best image texture measure, first-order variance calculated within a 15×15 moving window from the air photo. This measure predicted 54% of the variance in avian species richness. In a similar analysis, the standard deviation summary of first-order standard deviation calculated within a 51×51 moving window from a similar resolution air photo, explained approximately 56% of the variance in avian species richness in a Chihuahuan desert grassland-shrubland-pinyon-juniper study area in New Mexico [24]. First-order standard deviation and first-order variance are strongly correlated texture measures. Furthermore, we found that the standard deviation summary of first-order variance in a 51×51 moving window, the window size used by [24], was also moderately related to avian species richness (accounting for 42% of the variance). First-order texture measures derived from high resolution imagery exhibited strong correlation among extents (i.e., window sizes) [25]. Together, these findings suggest image texture derived from relatively fine-grained 1-m resolution remote sensing data can be useful for characterizing a surrogate of biodiversity [40], avian species richness, across broad spatial extents.

While NDVI has successfully predicted avian biodiversity patterns in other studies [31], it was not strongly associated with patterns of species richness at our study area. Field-measured vertical and horizontal vegetation structure performed better in explaining variation in species richness. Image texture calculated from 30-m resolution NDVI in areas with subtle changes in vegetation may characterize within-habitat variability related to avian species richness [31]. However, habitats that varied greatly in vegetation structure (e.g., savanna) occurred in a heterogeneous mosaic throughout our study area. Depending on the landscape context of a habitat patch, a moving-window analysis may have quantified pixel values from one habitat (e.g., woodland) into the texture values assigned on the outer edge of a sample point located in another (e.g., savanna). This may have masked the ability to quantify important vegetation structure heterogeneity (i.e., tree and shrub cover) relevant for species richness patterns. Thus, if habitats are structurally heterogeneous and distributed in a patchy mosaic, we suggest image texture from fine-grained imagery (e.g., 1-m resolution) may best characterize avian species richness across broad extents. If habitats are structurally heterogeneous, yet broadly distributed, we suggest texture from Landsat or imagery with similar resolution is appropriate for characterizing avian species richness.

Our study identified both opportunities and pitfalls of using image texture for predicting avian density and richness across broad spatial extents. In general though, models that are based on training data from one region may not have equal predictive power when applied to another area. For example, in southern Germany, predictive maps of the breeding distribution of Red Kite (*Milvus milvus*) were developed for the state of Bavaria and tested in the neighboring state of Baden-Württemberg [70]. Despite the proximity of the training and test sites, the predictive power of the model decreased, likely because of spatially autocorrelated data, and differences in environmental variables (e.g., elevation) between the two regions [70]. In our case, elevation was similar throughout our study area and there was no spatial autocorrelation affecting models of indicator species density or avian species richness. Yet, the habitat types vary greatly. The predictive maps of grasshopper sparrow and ovenbird generally captured the grassland and woodland areas outside of our training data sites well, which these birds use as breeding habitat (Fig. 5). These predictions were derived from good-fitting models (i.e., high *R^2^*) with relatively low prediction error. On the other hand, predictive maps for field sparrow and avian species richness were far noisier, either because the models were poor-fitting (e.g., field sparrow), or the prediction error was high (e.g., avian species richness). In general, using our approach, it is necessary to understand the strength of relationship between species density, or richness, and an image texture measure in addition to understanding the model prediction error for data from training sites. Furthermore, we suggest, when making predictions, that it is necessary to have a clear understanding of the habitat (i.e., environmental variables), including the observed variation of remotely sensed data, at both the training and test sites.

Field-based data collection methods are used by ecologists in order to understand plant and animal distributions. Though field-based data provide indispensable information, it is nearly impossible to collect such data rapidly and economically at broad scales. As land use pressures intensify and habitats continue to dwindle, conservation practitioners must apply efficient tools for prioritizing conservation. Our results suggest image texture can be useful data for understanding habitat quality for single species, and for characterizing a surrogate of biodiversity, avian species richness, across broad extents and therefore is valuable for management and conservation applications.

## Supporting Information

Figure S1 Pairs plots of two field-based measures of vegetation structure: vertical (foliage-height diversity) and horizontal (horizontal vegetation structure), the pixel values from an infrared air-photo (Air.photo) and a Normalized Difference Vegetation Index (NDVI) calculated from Landsat TM imagery, and two first order texture measures, entropy and variance, and one second order measure, contrast. The image texture measures were calculated in a 3×3 moving window from the air photo and summarized by the mean value (raw pixel values or image texture measures) in a 100-m radius circle surrounding each bird point count circle.
(TIFF)
