# Supplementary figures and images for: Image Texture Predicts Avian Density and Species Richness
Source: PLoS One. 2013 May 10;8(5):e63211. doi: 10.1371/journal.pone.0063211 (PMC3651168; doi:10.1371/journal.pone.0063211)

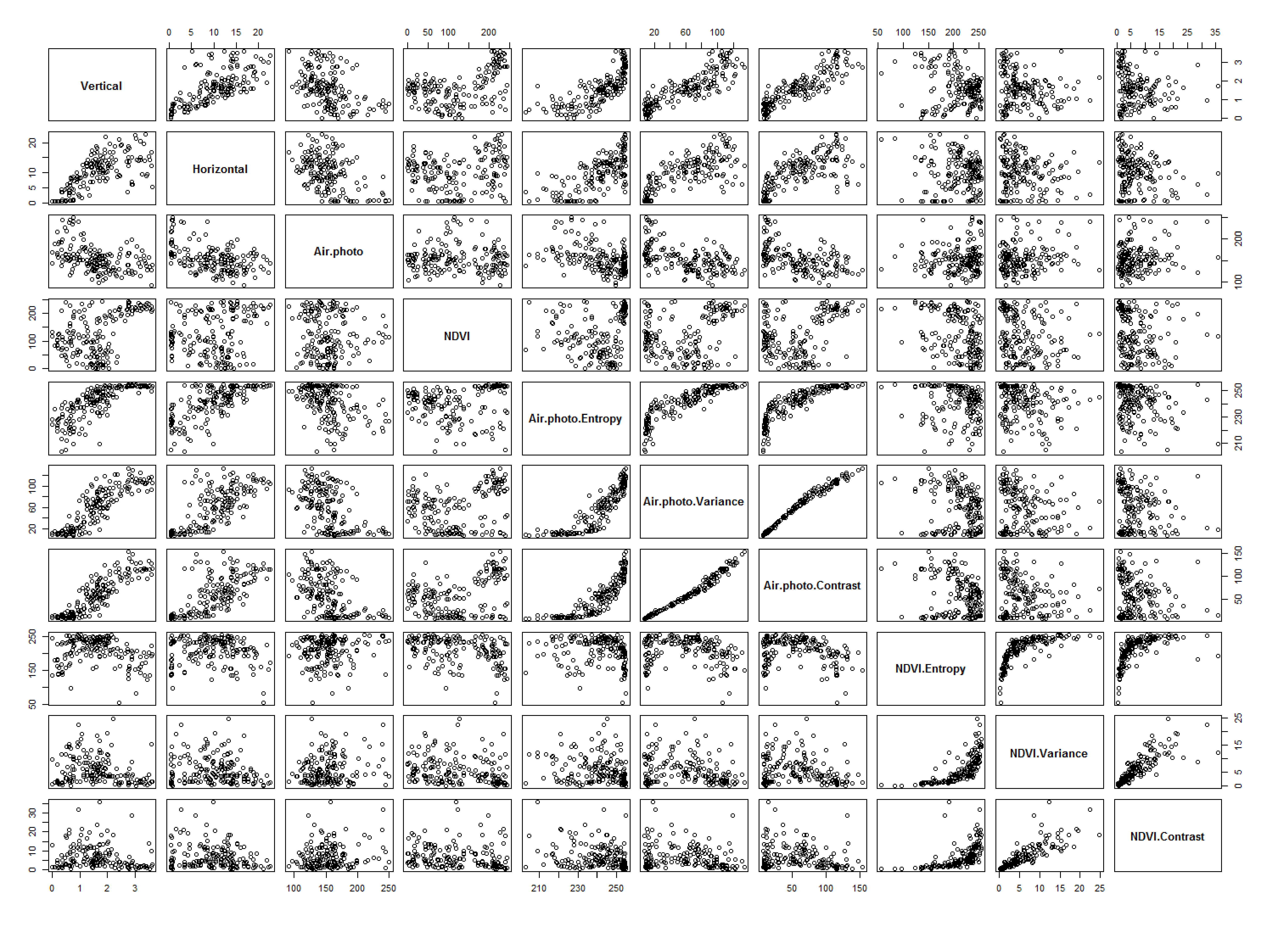

Supplement: Figure S1 — Pairs plots of two field-based measures of vegetation structure: vertical (foliage-height diversity) and horizontal (horizontal vegetation structure), the pixel values from an infrared air-photo (Air.photo) and a Normalized Difference Vegetation Index (NDVI) calculated from Landsat TM imagery, and two first order texture measures, entropy and variance, and one second order measure, contrast. The image texture measures were calculated in a 3×3 moving window from the air photo and summarized by the mean value (raw pixel values or image texture measures) in a 100-m radius circle surrounding each bird point count circle. (TIFF) [file pone.0063211.s001.tif]
